# A conception of integrated phased model combining sleep hygiene and stimulus control as an adult sleep education approach

**DOI:** 10.3389/fneur.2024.1513509

**Published:** 2024-12-18

**Authors:** Anastasiia D. Shkodina, Dmytro I. Boiko

**Affiliations:** ^1^Department of Neurological Diseases, Poltava State Medical University, Poltava, Ukraine; ^2^Non-governmental Organization “Institute of Sleep Disorders and Psychotraumatic Disorders”, Lviv, Ukraine; ^3^Department of Psychiatry, Narcology and Medical Psychology, Poltava State Medical University, Poltava, Ukraine

**Keywords:** sleep education, sleep disturbance, sleep hygiene, stimulus control, structured education

The prevalence of mental illnesses, particularly those associated with the impact of stress, is on the rise globally. Conditions like stress, depression, and anxiety are intricately connected to sleep and circadian rhythms through shared underlying mechanisms, underscoring the significance of sleep health as a critical public health concern (1, 2). Currently, cognitive behavioral therapy is considered the gold standard for the treatment of insomnia. Still, its widespread implementation has several obstacles, including limited public knowledge about the need for and function of sleep, the role of behavioral changes in improving sleep, and the lack of trained professionals (3, 4).

Amid Russia's full-scale invasion of Ukraine, the ongoing mental strain experienced by adults demands special attention. It is crucial to recognize that those facing chronic stress are a high-risk group requiring essential support and knowledge. The repercussions of prolonged stress and sleep disturbances can significantly impact overall health and wellbeing (5). The current lack of experts in the fields of sleep medicine and sleep neuropsychology highlights the urgent need for accelerated training in this field. This notion has been supported by modern research, emphasizing the effectiveness of comprehensive sleep education for the general population (6, 7).

Under such conditions, implementing group sleep education may be regarded as a beneficial intervention. Sleep education usually includes an overview of the functioning of the sleep-wake system, normative indicators of the regimen, their changes throughout life, information on various sleep disorders, a sleep hygiene checklist, and stimulus control (8). In October 2023, during the Third Module of the international specialized certificate program “Sleep. Stress. Psychological Trauma” orginized by the NGO “Institute of Psychotraumatic and Sleep Disorders” (Lviv, Ukraine) Malik Ait-Aoudia proposed a new concept of “somnoeducation,” which means training on sleep and its disorders in the process of psychotherapy and includes a thorough explanation of the problem and ways to correct it in the individual situation of the client. In general, somnoeducation can provide a solid foundation for restructuring and reframing the client's beliefs and can contribute to the ongoing therapeutic process. Two approaches of somnoeducation have been proposed: the approach of Malik Ait-Aoudia, when a separate psychotherapeutic session is held to educate the client and explain the main aspects of the sleep disorders and further treatment; and the approach of Oksana Voloshyna, who founded the field of Somnotherapy in Ukraine, when somnoeducation is carried out throughout the psychotherapist's work with the client on sleep disorders during each session (9).

At the same time, sleep education for the public is a fairly popular method of improving public health worldwide. This can be done through online courses, mailings, public lectures, training, and much more (10). A helpful tool for this type of training is often seen as a sleep hygiene checklist and the use of stimulus control. While sleep education has the potential to significantly enhance overall sleep quality worldwide, it is not widely utilized in practical applications and research. The majority of studies investigating the efficacy of sleep education have focused on children, teenagers, students, and older adults (11–13). Typically, sleep education includes recommendations for improving sleep and suggestions for eliminating factors that may disrupt sleep. Some clinicians do this by presenting these tips in a “desirable to add-desirable to remove” format, while others simply provide a checklist of recommendations.

Studies show that sleep education, which includes basic sleep hygiene and stimulus control, helps improve sleep quality and reduce symptoms of stress and anxiety. Such training programmes have shown positive effects even when delivered remotely (11). At the same time, a study of structured infant sleep education for mothers demonstrated that the educational intervention had a significant impact on mothers' knowledge over time, regardless of socio-demographic indicators, and is useful for developing healthy sleep habits in infants (14).

**Figure 1:**
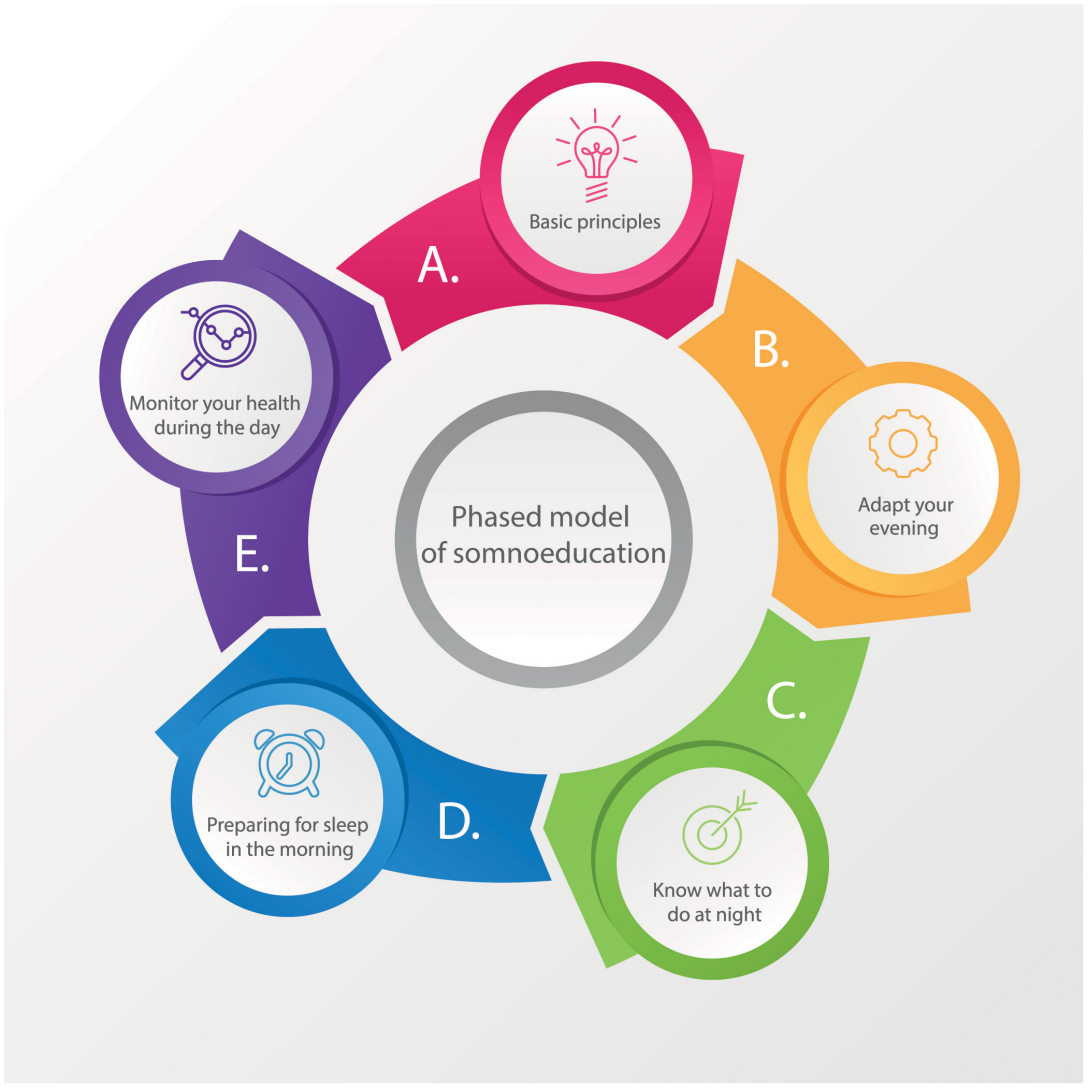
The phased model of somnoduction includes 5 stages that are presented to the patient step by step. This is followed by an assessment of the client's problems at this stage and education in the principles that relate to this particular block.

Based on our clinical experience, we have formulated a conception of integrated phased model of somnoeducation for edults that has garnered considerable interest among both clients and participants of sleep education events. The model consists of a combination of sleep hygiene recommendations and stimulus control therapy, which are traditionally used in cognitive behavioral therapy for insomnia and other behavioral therapies for sleep disturbances. All these recommendations are divided into five blocks: “Basic principles,” “Adapt your evening routine for falling asleep,” “Know what to do at night,” “Start preparing for sleep in the morning,” and “Monitor your health during the day” (Figure 1). The basic principles encompass guidance on sleep regularity and duration, the significance of associating the bed with sleep, and suggestions for creating a conducive sleep environment and also explanation of patients' sleep disorders themselves. The second set of guidelines offers advice on pre-sleep routines, such as establishing rituals, managing evening stimuli and behaviors, and potentially beneficial relaxation techniques. The third set provides tips for nighttime behavior in the event of difficulty falling asleep or frequent awakenings. The fourth section includes recommendations for starting the morning with a positive mindset and underscores the importance of natural light for sleep. The final section contains suggestions for daily behavior and habits that may impact an individual's sleep quality.

The sleep education using this model was presented by Anastasia Shkodina on September 16, 2024, during the training “Healthy sleep during war and techniques to improve sleep” in Poltava, Ukraine, attended by 48 people. According to the participants' survey, 40 people (83.3%) indicated that they strongly agreed that the material helped them improve their knowledge of physical health care, and another eight people (16.7%) indicated that they somewhat agreed with this statement. In addition, 39 participants (79.2%) strongly agreed that the information improved their knowledge of mental health care, and another 10 people (20.8%) somewhat agreed. In addition, 37 people (77.1%) strongly agreed that it increased their knowledge of the effects of stress on the body, nine (18.8%) somewhat agreed, and two (4.1%) chose the option “difficult to answer.”

Structuring sleep education throughout the day, as in a phased approach, offers significant benefits, as it allows information to be gradually and purposefully adapted to improve uptake and adherence. A structured approach to patient education is known to significantly improve patients' knowledge of their disease and help them retain this knowledge over time, as well as increase their adherence to therapeutic recommendations. Patients who receive structured education report higher levels of satisfaction with their healthcare, including communication with healthcare professionals and instructions (15, 16). A phased approach to education also promotes the development of autonomy and practical skills, which are key to health. For example, research shows that using a phased approach to educate patients with chronic diseases helps them to better learn self-care skills and adhere to necessary lifestyle changes (17). The effectiveness of phased education is that it lays the foundation for long-term behavioral change. This approach helps participants not to be overwhelmed by a lot of information at once, which is especially useful when working with patients who have limited resources for learning (18) or high levels of anxiety that often accompany sleep disorders. The use of a phased approach for sleep education allows us to align recommendations with periods of increased or decreased activity of body systems. For example, circadian rhythms determine the most effective periods for cognitive and physical activity and the most favorable periods for rest (19).

We recommend considering a structured approach to sleep hygiene and stimulus control education in future research to enhance consumer acceptance of this information. An integrated phase model may be beneficial to organize the training into different parts of the day to effectively guide the client through the advices. Specifically, the client can identify the specific time block in which they experience disturbances and concentrate on addressing it during therapy.
