# Supplementary material for: A conception of integrated phased model combining sleep hygiene and stimulus control as an adult sleep education approach
Source: Front Neurol. 2024 Dec 18;15:1513509. doi: 10.3389/fneur.2024.1513509 (PMC11688465; doi:10.3389/fneur.2024.1513509)
Supplement: Supplementary file 1 [file Data_Sheet_1.docx]

**Basic principles**

1. Explaining to the patient their sleep disorder, why it occurs and what it means for them, as well as possible ways to correct it.

2. Ensure regularity of sleep

Go to bed and wake up at the same time every day (+/- 1 hour), even on weekends and holidays. It will help adjust your biological clock and improve your sleep quality. Lack of sleep during the week also cannot be fully compensated for by sleeping longer on the weekend. While this sleep pattern will help you feel better, it won't fully compensate for the lack of sleep. It can also affect your biological clock, making it much harder to fall asleep at the right time on Sunday night and wake up early on Monday morning.

3. Remember that the bed is for sleeping

If you are having trouble sleeping, try not to use the bed for anything other than sleeping and having sex so that your body re-associates the bed with sleep. It is important because associating getting into bed with going to sleep will signal your brain that it's time to sleep.

4. Create a favourable sleep environment

4.1. Make your room dark. Use curtains or blinds to block out light. If you are bothered by light at night, wear a sleep mask. If you need light, use a dim light, such as a nightlight.

4.2. Keep the room humid and cool. Avoid extreme heat or dry air. It is known that sleep is associated with a decrease in temperature. It is better to sleep under a blanket in a cool room. At the same time, dry air can make breathing difficult and impair nighttime sleep. Use different methods of humidification as needed.

4.3. Make your sleeping place quiet. If noise bothers you, consider wearing earplugs or using soothing music or ‘white noise’ that turns off after 30-40 minutes.

4.4. The bed should be comfortable. The mattress, pillow and blanket should be comfortable and soothing.

**Adapt your evening routine for falling asleep**

1. You can develop your own rituals to remind your body that it is time to go to bed, for example, some people find it helpful to do relaxation or breathing exercises before bed each night or to sit quietly with a book, but avoid activities that may be stimulating. Wait until you feel drowsy or sleepy before going to bed. If you do not want to sleep at your usual time, try to relax.

2. A hot bath 1-2 hours before bedtime can be helpful because it raises your body temperature, making you feel drowsy when your body temperature drops again.

3. Stop using electronic devices 30 minutes before bedtime. Electronic devices, through which we access social media, news, etc., can contribute to the unexpected emergence of emotions, such as stress, excitement, and anger, which can prevent you from falling asleep. At the same time, bright light from electronic screens also disrupts the normal sleep-wake schedule by interfering with the natural daily cycle of light and darkness.

4. Practice relaxation techniques before bed, deep breathing and visualising calming and safe images. Muscle and breathing relaxation exercises do not lead to sleep immediately, but allow the body to slow down, calm down and prepare for sleep.

5. Have a light snack, but avoid heavy meals before bed. It is important not to feel hungry before bed, but a full stomach makes it difficult to sleep. The evening meal should be at least 2 hours before bedtime. Some people find that having a light snack before bed helps them sleep better. Drinking enough fluids during the day is also important for good sleep, but too much in the evening can be disruptive to sleep due to frequent waking up to use the toilet.

4.6. Some people lie awake in bed at night and are unable to switch their thoughts. If you have this problem, set aside ‘worry time’ in the evening. Use this time to think about what happened during the day and make plans and possible solutions for the next day.

**Know what to do at night**

1. The bedroom should be free of distractions. Remove or hide your watch out of sight so that you don't have to check the time constantly. Many people who have trouble sleeping tend to look at their watches too often. Frequent checking of the clock at night can wake you up (especially if you switch on the light to find out the time) and reinforce negative thoughts such as ‘Oh no, it's so late, I probably won't get any sleep again’ or ‘It's so early, I only got 5 hours of sleep, that's terrible’.

2. Do not force yourself to try to sleep, as this is likely to make you more alert. Sleep is not something you can force to happen. Rest is good - and it doesn't have to be sleep. Do not set yourself the expectation of insomnia, as this will increase your anxiety and only make it harder to fall asleep. If you are unable to fall asleep after 20-30 minutes or more (according to your own feelings), get up and do something calming or boring until you feel sleepy, then go back to bed and try again. Sit quietly on your bed with the lights off (bright lights will tell your brain it's time to wake up) or read something boring. Don't do anything too exciting or interesting because it will wake you up even more.

3. If you can't sleep because of annoying thoughts, remember that we can never turn off our minds. Our thoughts appear and are there all the time, so try to make them calmer. Create your favourite fantasy place. Or dream about your favourite holiday destination. If other thoughts come up, think about them for a moment and then try to gently replace them with calm thoughts.

**Start preparing for sleep in the morning**

1. Morning and early afternoon light will help you maintain a healthy sleep-wake cycle. Too much light in the evening can prevent you from feeling sleepy. Ensure you get enough natural light during the day. Try to spend at least 30-60 minutes in bright daylight in the morning. Surround yourself with bright light in the morning to make it easier to wake up.

2. If you find it difficult to wake up in the morning, develop rituals - a consistent routine that will signal to your body that a new day has begun.

**Monitor your health during the day**

1. Avoid caffeine in the afternoon. The effects of caffeine can last for several hours after ingestion. Caffeine is a stimulant whose effect is greatest during the first hour, but it takes up to 8 hours for the body to eliminate it. Caffeine increases the time it takes to fall asleep, the frequency of nighttime awakenings shortens the total sleep time, reduces REM sleep, and worsens the overall quality of sleep. Remember that caffeine is not only found in coffee.

2. Avoid alcohol of any kind for six hours before bedtime. Alcohol creates the illusion of falling asleep quickly and having a good sleep but negatively affects its structure. Sleep becomes fragmented and superficial, and breathing during sleep may be disturbed.

3. Do not smoke or use nicotine within two hours before bedtime. Nicotine also causes sleep to become more shallow. At the same time, smokers may wake up with the urge to smoke, which also disrupts the quality of sleep.

4. Exercise regularly. Exercising during the day can help you get a better night's sleep. However, intense exercise within 2-3 hours before bedtime can make it harder for you to fall asleep. Try doing a relaxing exercise before bed to help you fall asleep, such as yoga or relaxation exercises.

5. If possible, avoid napping during the day. If you must take a nap, keep it to 15-20 or 90 minutes before 3 pm. Naps can reduce nighttime sleep deprivation, but if you take a nap in the afternoon, you will have a harder time falling asleep in the evening.
